# The impact of covid-19 pandemic on pregnancy outcome

**DOI:** 10.1186/s12884-023-06098-z

**Published:** 2023-11-22

**Authors:** Roya Gholami, Nasrin Borumandnia, Elham Kalhori, Mahshid Taheri, Nahid Khodakarami

**Affiliations:** 1 Department of Midwifery, Faculty of Nursing and Midwifery, Tehran Medical Sciences, Islamic Azad University, Tehran, Iran; 2 Urology and Nephrology Research Center, Shahid Beheshti University of Medical Sciences, Tehran, Iran; 3 Iran Scientific Association of Midwifery, Tehran, Iran; 4 FBW Gynecology Plus, Ashford, South Australia Australia; 5 Men’s Health and Reproductive Health Research Center, Shahid Beheshti University of Medical Sciences, Tehran, Iran

**Keywords:** COVID-19, Pregnancy outcome, Preeclampsia, Gestational diabetes, Cesarean section, Preterm birth, NICU admission

## Abstract

**Background:**

The acute respiratory disease caused by the coronavirus (COVID**-**19) has spread rapidly worldwide yet has not been eliminated. The infection is especially deadly in vulnerable populations. The current studies indicate that pregnant women are at greater risk of getting seriously ill. Even though fetuses protect against disease, the additional finding showed that the COVID-19 pandemic could increase fetal and maternal morbidities. In a situation where COVID-19 and new strains of the virus are still not controlled, scientists predicted that the world might experience another pandemic. Consequently, more research about the effects of COVID-19 infection on pregnancy outcomes is needed. This study aimed to compare the pregnancy outcomes of Iranian pregnant women in the first year of the pandemic with the previous year.

**Methods:**

This prospective cross-sectional study was performed to compare the pregnancy outcome during the COVID-19 pandemic among Iranian pregnant women who gave birth during the pandemic and one year before the pandemic (2019–2020 and 2020–2021). The sample size was 2,371,332 births registered at hospitals and birth centers platforms. The studied variables include stillbirth, congenital anomaly, birth weight, preeclampsia, gestational diabetes, cesarean section, ICU admission, mean of the gestational age at birth, preterm births, NICU admission, neonatal mortality and the percentage of deliveries with at least one complication such as blood transfusion and postpartum ICU admission. Analyzing data was done by using SPSS version 25 software.

**Results:**

We found statistical differences between pregnancy and birth outcomes during the COVID-19 pandemic compared to one year before. The risk of preeclampsia, gestational diabetes, cesarean section, preterm birth and NICU admission were clinically significant. Also, there was a significant decrease in mean gestational age.

**Conclusion:**

The COVID-19 pandemic has affected the pregnancy outcome by increasing morbidities and complications during pregnancy, birth, and postpartum. In addition, extensive quarantine outbreaks disrupted the healthcare system and hindered access to prenatal services. It is necessary to develop preventive and therapeutic care protocols for similar pandemic conditions.

## Background

Since the earliest report of the epidemic and unknown cause of pneumonia in Wuhan, China, in December 2019 [1] and identifying the novel coronavirus as its causative agent [2], now called SARS-CoV-2. According to a World Health Organization report, over 651 million were infected with (COVID-19) and over 6 million associated deaths related to the COVID-19 global outbreak [3]. The same data reported 7,560,629 registered cases and 144,672 related deaths in Iran [4]. Acute disease caused by the coronavirus (COVID-19) has spread rapidly and has not been controlled yet. The COVID-19 pandemic crisis threatens the healthcare system, lifestyle, social structures, the global economy, and all aspects of life in the world [5, 6].

COVID-19 is particularly deadly in vulnerable populations [7]; the studies indicate that pregnant women are at greater risk of getting seriously ill, but the transmission rates from mother to fetus are rare [8]. Pregnant women and their fetuses are considered a high-risk population during the outbreak of infectious diseases because physiological and mechanical changes in pregnancy generally increase susceptibility to infection. In addition, the predominance of the T-helper 2(Th2) system to protect the fetus in pregnancy makes the mother more vulnerable to viral diseases, which are more inhibited by the system[9]. Since the beginning of the coronavirus epidemic, pregnant women have been introduced as a vulnerable group, and special instructions have been published worldwide to protect them from contracting the disease [5]. Although studies have shown that the manifestations and consequences of COVID-19 illness in pregnant women are similar to those in their non-pregnant cohorts, hospitalization of infected cases in the intensive care unit increased among pregnant women, indicating a lower threshold for interventions for pregnant women [10]. In addition, the pandemic affected lifestyle, the quality and quantity of prenatal care, and increased chronic anxiety.

Additionally, COVID-19 might be at increased risk for other adverse outcomes, such as elective cesarean Sect. [11–13]. The impact of the COVID-19 pandemic on pregnancy was not limited to severe respiratory disease and maternal mortality; the nationwide quarantine outbreak might disrupt essential maternal child health services [14, 15]. A meta-analysis study revealed an overall increase in the chances of stillbirth, maternal death, rupture of ectopic pregnancy, and maternal depression during the pandemic. The results have shown a significant variation in the high and low financial resources and the severity of the disease impact in global regions [16]. Likewise, fetal outcomes worldwide have worsened [17, 18].

In a situation where COVID-19 and new variants detected through the epidemic have not been wholly controlled, scientists predicted that the planet might experience another pandemic. Therefore, an evidence-based approach is needed to improve maternal and newborn care quality, infection prevention, and control against pandemics. Therefore, this study aimed to compare the pregnancy outcomes of Iranian pregnant women within the first year of the pandemic with the previous year.

## Material and methods

The Iran Ministry of Health reported the first confirmed cases of COVID-19 in Iran on February 30, 2020; through a retrospective cross-sectional study, we studied the impact of the COVID-19 pandemic on pregnancy outcomes among all the pregnancies that terminated within the first year of the COVID-19 pandemic, and one year before the pandemic (February 2019 to February 30, 2020, and from March 1, 2020, to March 1, 2021). The desired information was structured data from a preexisting information extraction from all hospitals' birth registry platforms, which typically collected data related to pregnancy and birth [A1]. This study's sample size was 2,371,332 births registered during two years, including 1,221,333 births in 2020 and 1,149,999 births in 2021 (all pregnancy termination with and without COVID-19 infection). To determine the COVID-19 pandemic effect on pregnancy-related outcomes, we evaluated variables namely: Stillbirth (a baby with no vital sign delivered at or after 24 weeks), Congenital Anomaly, Birth Weight, Preeclampsia, Gestational Diabetes, Cesarean Section (Considering that the database did not allow extrapolation of data on the urgency of cesarean section or operative vaginal delivery, the variable of cesarean included emergency and elective cesarian section. The Variable of Vaginal Delivery included both normal vaginal delivery and operative vaginal delivery involving the application of forceps or a vacuum*)*. ICU admission, mean of the Gestational Age at birth, Preterm Births (extremely preterm (less than 28 weeks), very preterm (28 to 32 weeks) and moderate to late preterm (32 to 37 weeks), NICU admission, Neonatal Mortality and the percentage of deliveries with at least one complication like blood transfusion and postpartum ICU admission. Birth weight z-scores were calculated to adjust the birth weight for gestational age; the third-degree polynomial function was fitted to the 50th centile for the estimated fetal weight using World Health Organization fetal growth charts, and z-scores were calculated between 24 and 29 + 6 weeks gestation. Centile limitation was considered by using z-scores. Variables were defined according to clinical diagnosis registered at the hospital's data pool. The research ethics committee of the Shahid Beheshti University of Medical Sciences approved this study".

### Statistics analysis

After importing the data from the Excel platform to the SPSS version 25, we cleaned up the data, Set the KPIs, omitted useless data, and built a data management roadmap for data analysis. We used descriptive and inferential statistics to compare pregnancy outcomes among births between the two years of collected data (before and after the COVID-19 pandemic). Statistical significance levels by *P* < 0.05 were all two-sided. We used pooled standard deviation by considering Cohen's d Effect Size (ES) for Standardized differences between the two groups (Table 1).

**Table 1:** Comparison of pregnancy out come before Covid-19 Pandemic and during Pandemic era

Variables		Before pandemic era		During pandemic era		*P*-value	Effect size (${\mathbf{P}}_{\mathbf{d}\mathbf{u}\mathbf{r}\mathbf{i}\mathbf{n}\mathbf{g}\mathbf{C}\mathbf{o}\mathbf{v}\mathbf{i}\mathbf{d}19}$ -${\mathbf{P}}_{\mathbf{B}\mathbf{e}\mathbf{f}\mathbf{o}\mathbf{r}\mathbf{e}\mathbf{C}\mathbf{o}\mathbf{v}\mathbf{i}\mathbf{d}19}$)	Effect size per million
		**N**	**P**	**N**	**P**			
**Preeclampsia**	**Yes**	**25,188**	**2.1%**	**26,710**	**2.4%**	** < 0.001**	**2.36–0.26 = 2.10**	**2650**
	**No**	**1,176,833**	**97.9%**	**1,104,864**	**97.6%**			
**C/S among first gravida women**	**Yes**	**601,612**	**50.1%**	**623,158**	**55.1%**	** < 0.001**	**55.07–5.019 = 50.05**	**50,200**
	**No**	**600,409**	**49.9%**	**508,416**	**44.9%**			
**Preterm birth**	**Yes**	**102,862**	**8.6%**	**99,266**	**8.8%**	** < 0.001**	**8.77–0.21 = 8.56**	**2150**
	**No**	**1,099,159**	**91.4%**	**1,032,308**	**91.2%**			
**Neonates hospitalized in the NICU**	**Yes**	**92,370**	**7.6%**	**90,898**	**7.9%**	** < 0.001**	**7.9–0.34 = 7.56**	**3411**
	**No**	**1,128,963**	**92.4%**	**1,059,101**	**92.1%**			
**Gestational diabetes mellitus**	**Yes**	**54,259**	**4.5%**	**61,661**	**5.4%**	** < 0.001**	**5.45–0.93 = 4.51**	**9352**
	**No**	**1,147,762**	**95.5%**	**1,069,913**	**94.6%**			

This table shows the variables that their changes were statistically significant between before and during pandemic era

## Results

Our baseline results focused on comparing maternal and neonatal outcomes of all pregnancies during the COVID-19 pandemic and the year before. The maternal age less than 18 years old age, was excluded. Due to inaccessible whole data related to pregnant women diagnosed with a positive RT-PCR for COVID-19, evaluating the difference between COVID-19 positive cases and controls was not the aim of this study.

### Maternal outcome

#### Birth complications

We did not find a significant relationship between the COVID-19 pandemic and deliveries with at least one birth complication. In addition, the need for mother blood transfusion was not statistically significant during the COVID-19 era and the year before.

#### ICU hospitalization

Five thousand four hundred forty postpartum women were hospitalized in ICU for any reason before the COVID-19 pandemic era, and 5520 during the pandemic era (including COVID-19 positive cases), 1.47% increase during the pandemic. Considering the ratio of births in the previous year to births during the COVID-19 pandemic, the risk of ICU hospitalization during the postpartum period was higher in the COVID-19 pandemic era; however, it was not statistically significant.

#### Preeclampsia and gestational diabetes

**Fig. 1:**
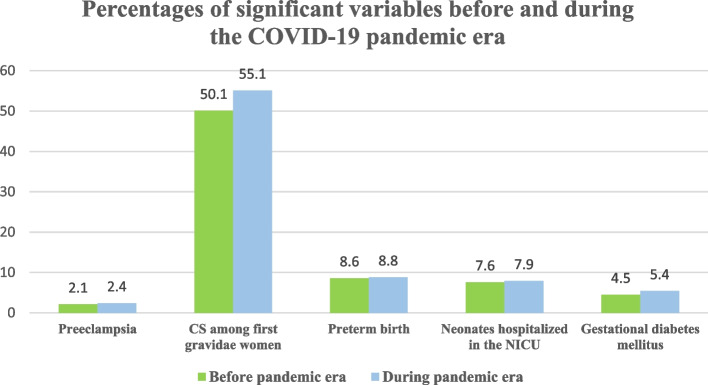
Shows the percentages of significant variables before and during the COVID-19 pandemic era

Preeclampsia and gestational diabetes mellitus increased significantly during the pandemic. (Fig. 1 and Table 1) Cesarean section delivery.

The overall cesarean section (CS) rate was 56% in the pandemic era and 54% in the year before the pandemic[A1]. The rate of CS among first gravidae women was 55.1 in the pandemic era and 50.1 in the year before. There was a steady rise in the cesarean section rate over time, with an average increase of 2% per calendar year and 5% for prim parous. Adjusting for this increase, the risk of cesarean section remained significantly higher during the COVID-19 pandemic era (Fig. 1 and Table 1).

### Neonatal outcome

#### Stillbirth and neonatal mortality

**Fig. 2:**
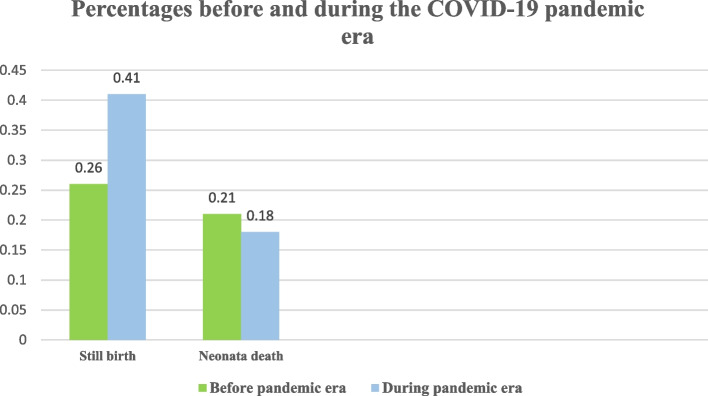
Shows the percentages of still birth and neonate death before and during the COVID-19 pandemic era

The number of stillbirths was 9565 in 2020 and 9780 in 2021 (0.26% and 0.41%). There was not a significant change in the risk of stillbirth (OR 0.78, 95% CI 0.51–1.20, *P* = 0.26) or neonatal mortality (OR 0.89, 95% CI 0.54–1.47, *P* = 0.64) in the COVID-19 era compared to the pre-COVID-19 era (Fig. 2).

#### Preterm birth

Forty-two deliveries were excluded from gestation analysis as they had an uncertain gestation age at the birth or more than 42 weeks. There was a significant rise in the preterm birth rate during the pandemic, from 8.6% to 8.8%. (Fig. 1 and Table 1).

We found significant changes in the risk of moderate to late preterm birth (32–37 weeks) compared to the COVID-19 pandemic era and no significant difference at less than 32 weeks (*p* < 001).

#### Birth weight

One hundred twenty samples were excluded from the birth weight analysis due to missing birth weight or gestation at the birth of fewer than 20 weeks.

The mean birth weight was 3,143 g ± 554 g, equating to a mean birth weight z-score of -0.47 ± 1.60 compared to the WHO fetal growth charts. Even though there were no significant changes in birth weight z-score over time (*P* = 0.54), there was a trend towards an increase in z-score in the COVID era (Mean difference 0.05, 95% CI 0.00–0.07, *P* = 0.059) compared to the pre-COVID pandemic.

#### Gestational age

Seventy-three newborns were excluded from the analysis of the mean age because of the invalid data.

The mean gestational age was 38.3 weeks ± 2.1 before and 38.2 weeks ± 2.1 during the pandemic. There was a significant decrease in gestational age over time (*P* < 0.001).

#### Congenital anomaly

The number of babies born with a congenital anomaly at birth was 6240 in the COVID-19 era and 5571 in the years before the pandemic (OR 0.76, 95% CI 0.53–1.20, *P* < 0.24).

#### NICU hospitalization

Neonatal Intensive Care hospitalization (NICU)was significantly higher than before the pandemic era (*P* < 001). (Fig. 1 and Table 1).

## Discussion

We found that the risk of preeclampsia and gestational diabetes in the pandemic era was higher than before the COVID-19 pandemic. In addition, during the pandemic era, there was a considerable increase in the Cesarean Section rate among nulliparous women for elective or maternal/fetus indications. Also, we observed a significant effect of the pandemic on overall preterm birth, ill newborns who need NICU hospitalization, and the mean gestational age decreased significantly.

Several studies claim that coagulation disorders and intravascular clot formation have been observed in severe cases of COVID-19 in non-pregnant people [19]. This virus disrupts the function of the endothelial system in direct or indirect ways, leading to severe inflammation and inappropriate antiviral responses [20, 21]. Also, it has been seen that COVID-19 causes specific vascular pathology, similar to the changes that occur in preeclampsia during pregnancy [22]. We found a specific relationship between the infection with COVID-19 and the risks of severe preeclampsia. There are limited studies on this field, but our findings agree with Papageorghiou et al. study[23].

Severe acute respiratory syndrome coronavirus 2 (SARS-CoV-2) binds to angiotensin-converting enzyme 2 (ACE2) receptors, expressed in critical metabolic organs and tissues, including pancreatic beta cells and adipose tissue. Thus, it can cause ketosis-prone diabetes, lead to glucose metabolism disorder, and destroy pancreatic beta cells [24]. This mechanism is the cause of diabetes mellitus development in patients with COVID-19 [25]. The covid-19 can also cause an autoimmune attack on pancreatic islet cells due to abnormal immunity [26]. On the other hand, it has been widely proven that reducing physical activity during pregnancy increases the risk of gestational diabetes mellitus (GDM). The GDM incidence rate was significantly higher during the COVID-19 pandemic. Liu et al. [27] and V Zanardo et al. [28] findings showed that the COVID-19 *pandemic* negatively affected *GDM* prevalence during pregnancy compared to the COVID-19 pandemic.

Although cesarean section (CS) rates in Iran increased over time [29], this study showed an increase of 2% compared to a year before. This study showed that the overall CS rate increased significantly compared to one year before. The physicians elected elective termination of pregnancy to aid the management of maternal COVID-19 disease or as a preventive issue to protect the mother and neonate from unwanted COVID-19 complications [30, 31], and pregnant women choose elective pregnancy termination by CS to give birth as short as possible because they do not have other choices such as home birth, home birth is not common in Iran and midwives do not have permission to give birth at home except in emergency cases.

As Debrabandere et al. found a significant rise in CS deliveries during the pandemic, our findings agree with this study [32]. Zhang et al., in a survey of non-infected women in 9 Chinese cities during the COVID-19 lockdown period, found a significant increase in the rate of cesarean deliveries [33]. However, the benefit of the CS or elective termination of pregnancy during the pandemic is unclear. Li et al., reported no evidence of any difference between the CS rate before and during the pandemic era in Wuhan [34]. However, the benefit of the CS or elective termination of pregnancy during the pandemic is unclear. Considering the short-term and long-term complications of cesarean section for the mother and the baby [35], it is not known that CS was beneficial during the pandemic. Therefore, additional good-quality studies are needed; in Parallel, Iran's health system and national media should consider childbirth education and Public awareness regarding the advantages of physiological birth.

We found a significant association between infection with COVID-19 and the mean gestational age, preterm birth rate, and NICU admission, as infected women's pregnancy termination was earlier than those without infection. These women's babies experienced a higher rate of preterm birth and NICU admission, consistent with reports of previous studies that found COVID-19 infection to be a risk factor for lower gestational age and higher preterm birth and NICU admission rate [27, 36, 37]. However, some studies reported that COVID-19 infection was associated with lower preterm birth and NICU admission rates during the pandemic lockdown [18, 38–40]. On the other hand, some authors believe there is no link between COVID-19 infection and preterm birth [41]. These events could be related to the increased preeclamptic pregnancies during the COVID-19 pandemic, although SARS-CoV-2 infection can lead to exaggerated systemic inflammatory responses, contributing to premature birth pathogenesis[36]. It seems that due to the experts' belief, the priority of early pregnancy termination is the subsequent treatment of COVID-19; even in mild cases of the disease, the rate of cesarean delivery and, as a result, preterm delivery has increased [42], differing to the studies that shown as a result of the lockdowns during the covid-19 era and reduced work hours decreased physical or emotional stress for pregnant women working outside the home. It reduced the time of being exposed to air pollution, and preterm labor was decreased [43].

We observed that stillbirth, congenital anomaly, and neonatal mortality rates did not vary before and during the pandemic. Additionally, the mean birth weight did not change remarkably.

There was no significant effect of the COVID-19 pandemic era on rates of deliveries with birth complications, need for mother blood transfusion, and ICU hospitalization overall.

Our study has some limitations. One of these limitations was the lack of access to precise statistics on maternal mortality and pregnant women with COVID-19 during the research period. The only available data were a limited number of articles from regional studies. So, these components were not mentioned in the study due to the unreliability of the statistics.

Despite some limited aspects of this population study, it may have enhanced the accuracy of the analysis. In addition, this study is unique because it analyzes the entire population of pregnant women who have given birth in any Iranian hospitals during this period.

This study showed that the pandemic is not having a remarkable effect on maternal outcomes in labor and delivery. However, an increased rate of CS delivery, preterm birth, and NICU hospitalization can be caused by fear of consequences or lack of sufficient knowledge in this field.

While we found an increased cesarean section rate among Iranian pregnant women, there seems to be significant global variation in obstetric practice that has changed during the worldwide pandemic.

COVID-19, like other crises such as war and natural disasters, has profoundly affected clinical practice due to its sensitive nature. Jordan sibeoni's study results present how Counseling and Psychological Clinical Services practice was disorganized by the pandemic and the working conditions that accompanied it, especially the loss or impediments of many of its ordinary or usual features: lived-time, lived space, colleagues, sensory qualities, embodied encounters, and connections with patients [44, 45]. Future evidence-based assessment designs and innovative approaches to COVID-19 effect on pregnancy and birth management can be implemented because COVID-19 will likely pose a challenge to maternal childcare systems in the future, and we will have to find ways to manage patients with COVID-19 while not neglecting non-infected pregnant women. Studies could help to improve a pandemic crisis management model for directing clinical practice.

### Study limitation

The strengths of this study were appropriate sample size, reliable data related to the study goals, and low missing data.

COVID-19 is an essential factor affecting maternal mortality rates. One of this study's limitations was the lack of access to precise statistics on pregnant women with COVID-19 during the research period and maternal mortality rates during the pandemic era and limited access to part of the data and information related to pregnant women who were infected or were hospitalized for Covid-19 as well, So, these components were not mentioned in the study due to the unreliability of the statistics.

## Data Availability

The datasets generated and analyzed during the current study are not publicly available due to national policy considerations. However, they are available from the corresponding author on reasonable request after providing an administration process.
